# Supplementary material for: Tit wit: environmental and genetic drivers of cognitive variation along an urbanization gradient
Source: Anim Cogn. 2025 Jul 3;28(1):56. doi: 10.1007/s10071-025-01962-1 (PMC12226638; doi:10.1007/s10071-025-01962-1)
Supplement: Supplementary file 1 — Supplementary file1 (PDF 959 KB) [file 10071_2025_1962_MOESM1_ESM.pdf]

Supplementary Materials

Title:

Tit wit: environmental and genetic drivers of cognitive variation along an urbanization gradient

Authors:

Thompson MJ*[1,2], Gervais L*[1,3], Bharath D[1], Caro SP[1], Chaine AS[3], Perrier C[4,5], Réale D[2], & Anne Charmantier[1]

*Joint first author

Affiliations:

[1]Centre d'Ecologie Fonctionnelle et Evolutive, Univ Montpellier, CNRS, EPHE, IRD, Montpellier, France

[2]Département des sciences biologiques, Université du Québec à Montréal, 141 Avenue du Président-Kennedy, Montréal, QC H2X 1Y4, Canada

[3]Station d'Ecologie Théorique et Expérimentale du CNRS, UAR 2029, Moulis, France

[4]UMR CBGP, INRAE, CIRAD, IRD, Institut Agro, Université Montpellier, Montpellier, France

[5]Centro Agronómico Tropical de Investigación Y Enseñanza (CATIE), Turrialba, Costa Rica

Corresponding Author:

Megan J. Thompson (thompsonjoymegan@gmail.com)

Supplementary methods:

Statistical power to estimate V_A in the animal model

To assess the statistical significance of the estimated additive genetic variance (V_A) in our real dataset, we constructed a null distribution through a simulation-based approach using the *squid*R package (Allegue et al. 2017). This methodology allows us to determine whether the observed V_A could have arisen by chance under the null hypothesis that $V_A = 0$. We generated 500 independent datasets using *SimSquid*, ensuring that the additive genetic variance (V_A) was set to 0 in each case, that V_{PE} was set to the observed V_A+V_{PE} estimated in Table 2 of the main text, and V_R was set to V_R estimated in Table 2 of the main text. In addition, we simulated the same fixed effects using the effect sizes estimated in our animal model (Table 2). Hence, the simulated dataset mimics the structure of the real dataset while eliminating genetic effects, providing a baseline expectation under the null hypothesis. For each simulated dataset, we applied the same linear mixed model as described in the main text. This model allowed us to estimate V_A in each case, ensuring comparability between simulated and real data results. To summarize the null expectation of V_A, we extracted the median V_A estimate from each of the 500 simulated datasets. This collection of 500 medians forms the null distribution, representing the range of V_A values that would be expected under the assumption that $V_A = 0$ (visualized in Figure S2a). We estimated statistical power as the percentage of the posterior distribution of observed V_A estimates that were greater than the median value of the null V_A distribution ($V_{Aobs}>V_{Anull}$; Pick et al. 2023). Our simulation analysis demonstrates that our dataset has enough statistical power (87.9%) to detect additive genetic variance different from zero for the number of errors (visualized in Figure S2b).

Censored regression approach

Trials of the motor detour task ended if birds had not found the exit within 180 seconds (22% of observations for common garden birds). To evaluate whether our results were robust when including all common garden individuals (those that both escaped and did not escape the task during the trial period), we fitted a Bayesian censored multilevel generalized regression with the brms package in R. These models allow inclusion of censored events of interest that did not occur during a defined study period; in this case individuals in the common garden with maximum latency values (180 seconds) are censored in the model since we don't know their true number of errors or latency to escape values. We fitted models using the same fixed and random effects as described for models 2 and 4 under the "Statistical approach" section of the main text. Results (see Table S2) were qualitatively similar to those presented in Table 1.2 of the main text where unsuccessful individuals were excluded from the analysis.

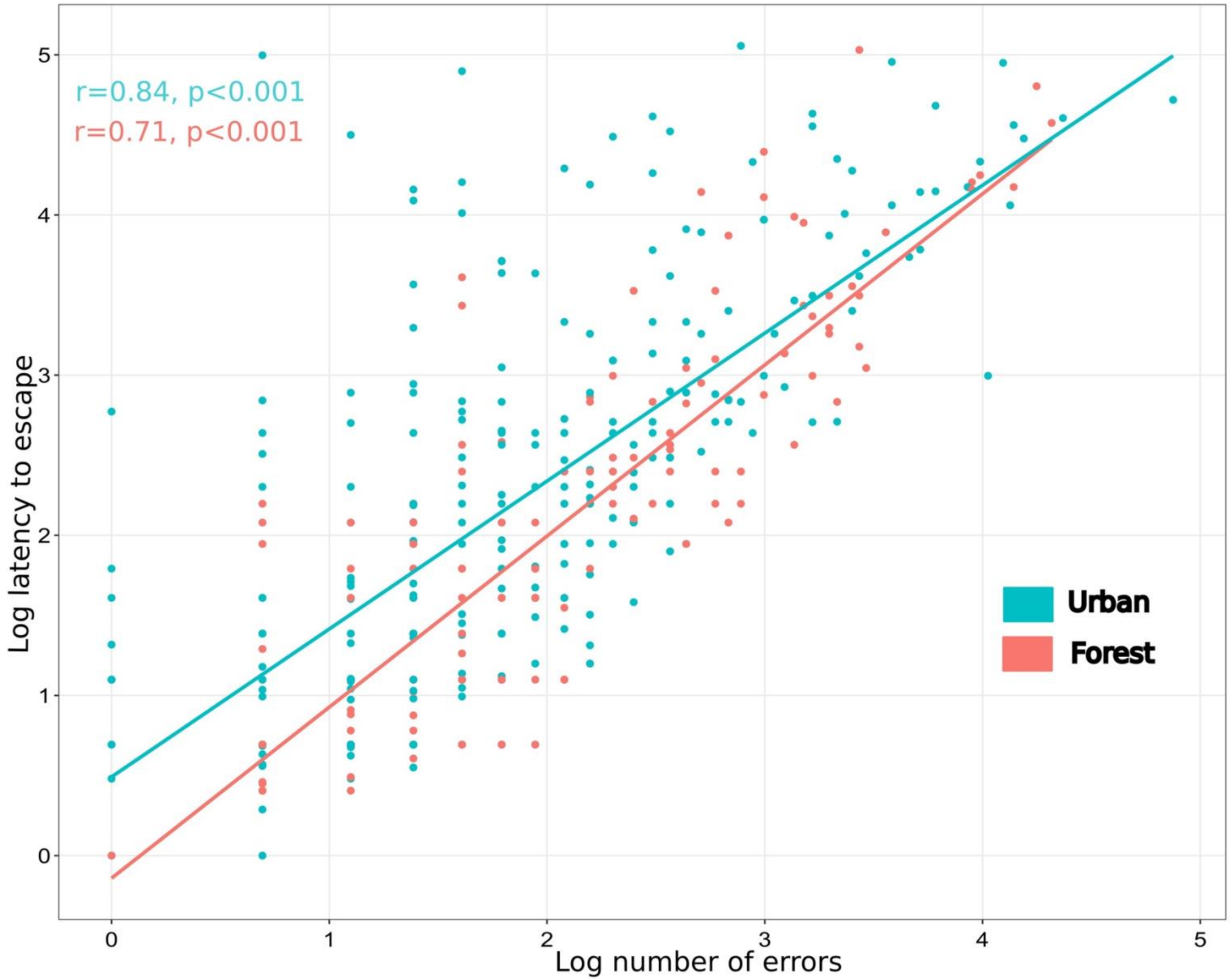

Fig.S1 Phenotypic correlation between the log-transformed latency to escape and the log-transformed number of errors for the urban population (blue) and the forest population (red). The correlation coefficient (*r*) and its associated p-value (both < 0.001) are shown.

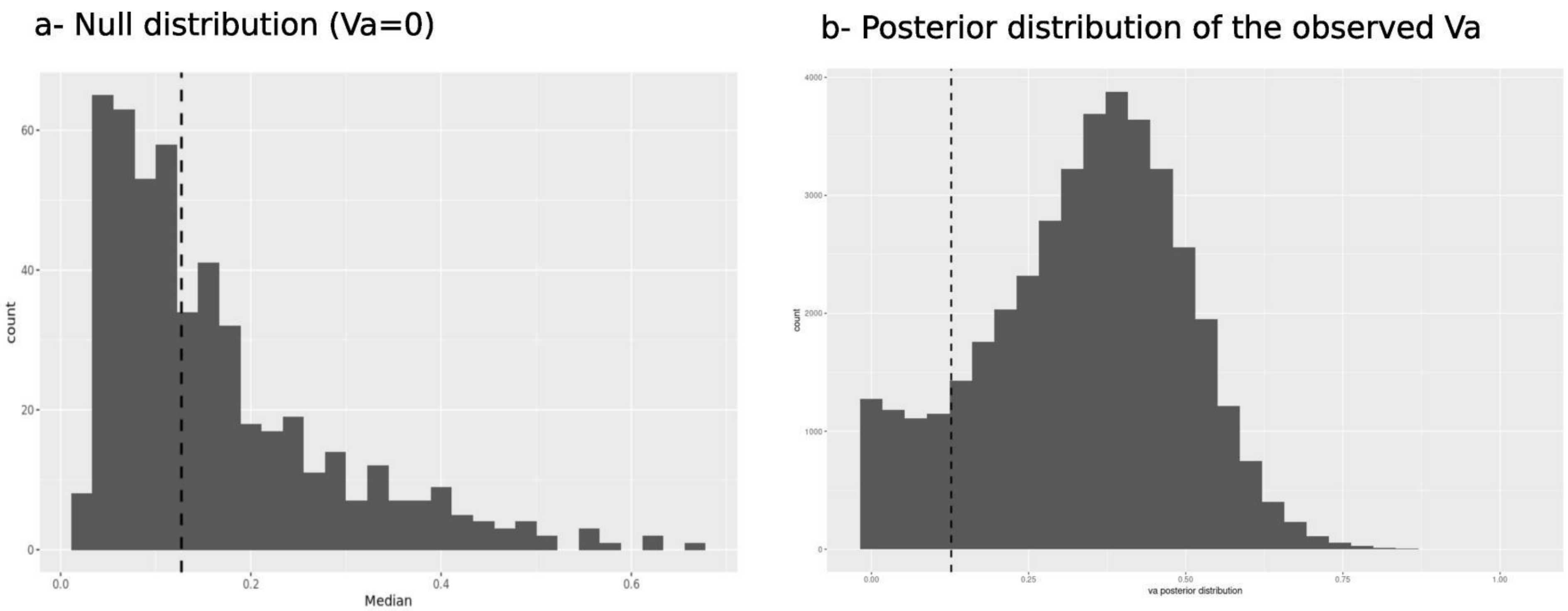

Fig.S2 Our simulation indicates a power of 87.9% to detect additive genetic variance different from zero for the number of errors during the task, as 87.9% of the posterior distribution of the observed V_A (shown in panel b) lies above the median of the null distribution (indicated by black vertical hashed line; shown in panel a). Null distribution (V_A=0) was generated from 500 simulation iterations.

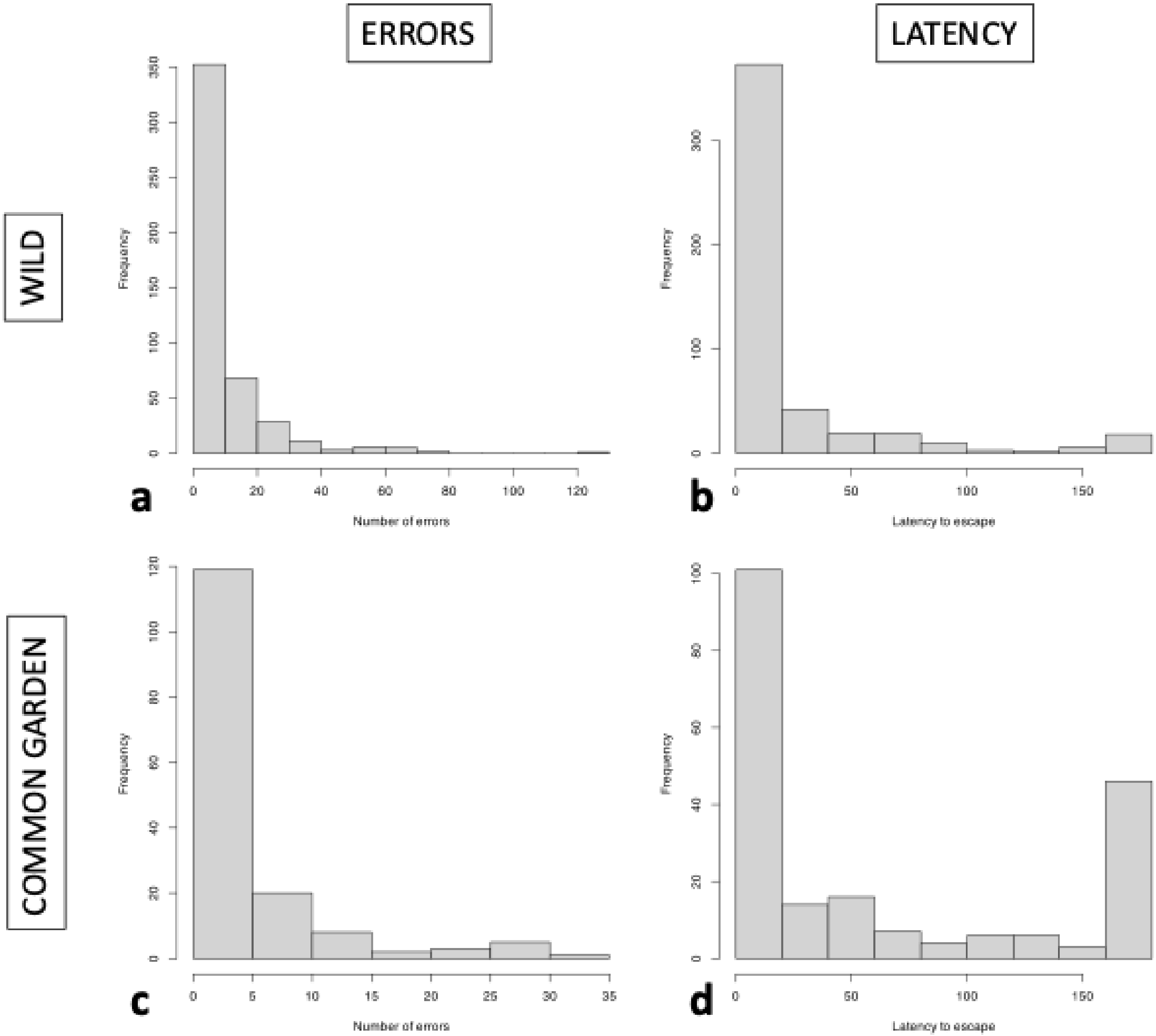

Fig.S3 Distributions of the number of errors (a & c) and latency to escape the cage (b & d) in the motor detour task administered in wild (top panel) and common garden (bottom panel) contexts.

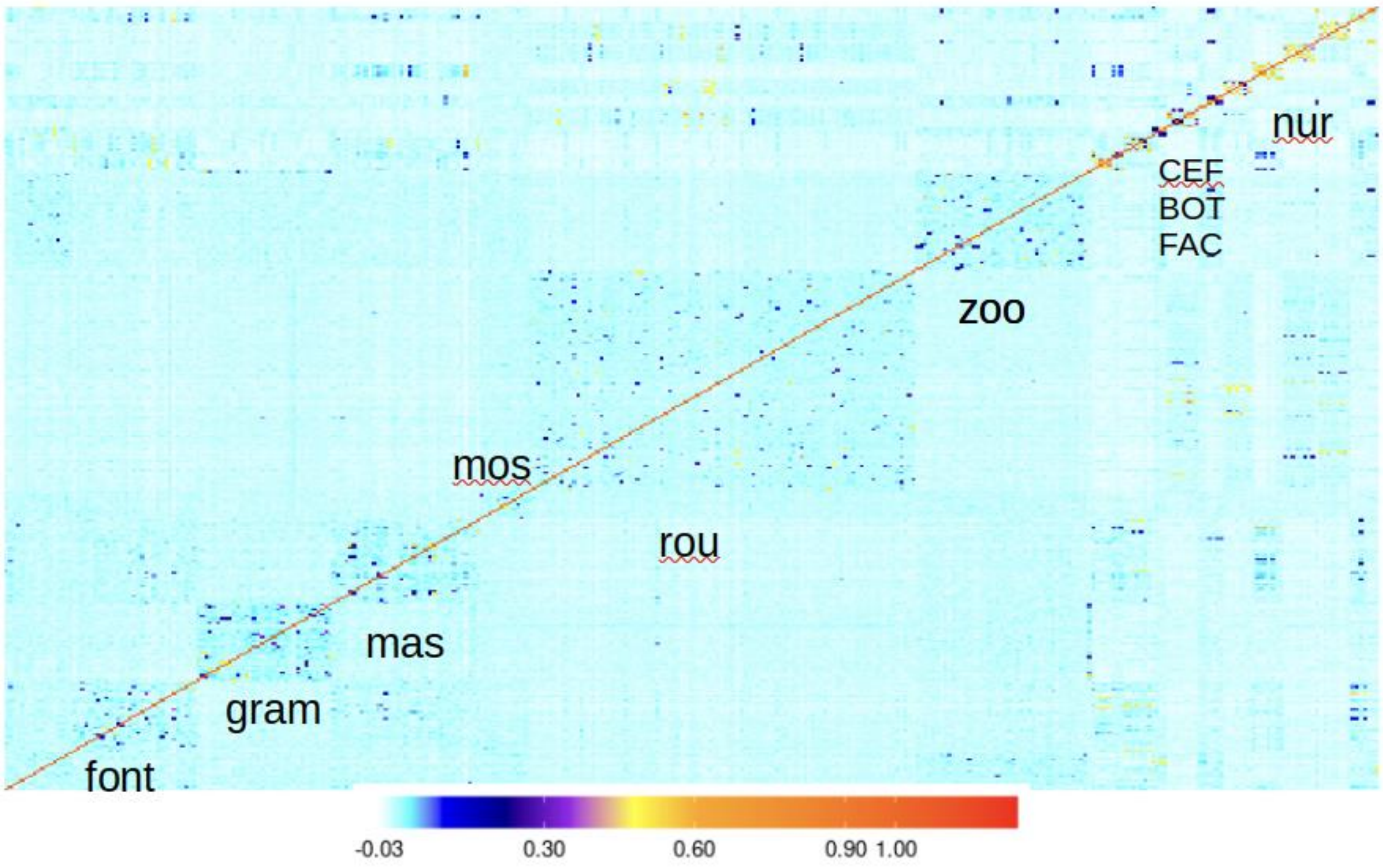

Fig.S4 Genomic relatedness heatmap, with relatedness represented by a color gradient ranging from cold colors (blue with low relatedness) to hot colors (red with high relatedness). The names of study sites and contexts are labeled in black (urban: font, gram, mas, mos, zoo, fac, cefe, bot; forest: rou; common garden birds: nur).

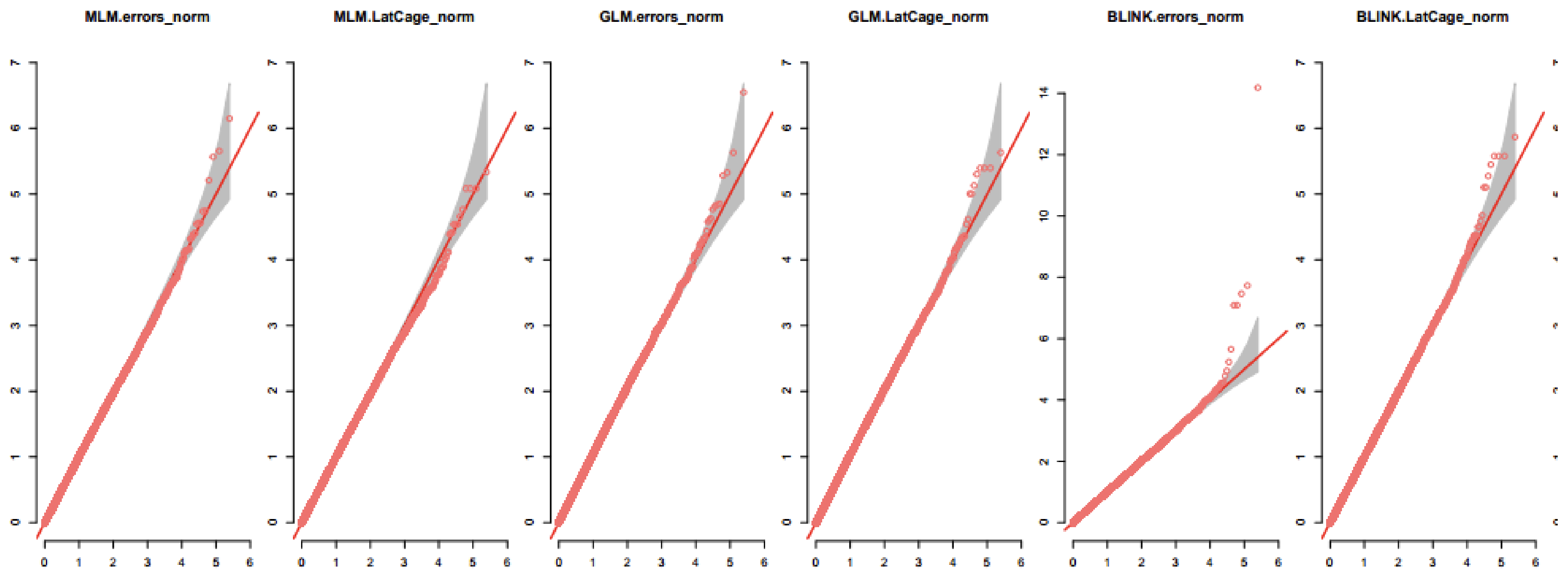

Fig.S5 Quantile-quantile (QQ) plots of *P*-values for the different GWAS models: MLM, GLM and BLINK for number of errors (errors_norm) and latency to escape the task (LatCage_norm). The y-axis is the observed negative base 10 logarithm of the *P*-values and the x-axis is the expected observed negative base 10 logarithm of the *P*-values under the assumption that the *P*-values follow a uniform [0,1] distribution. The grey surface shows the 95% confidence interval for the QQ plot under the null hypothesis of no association between the SNP and the trait.

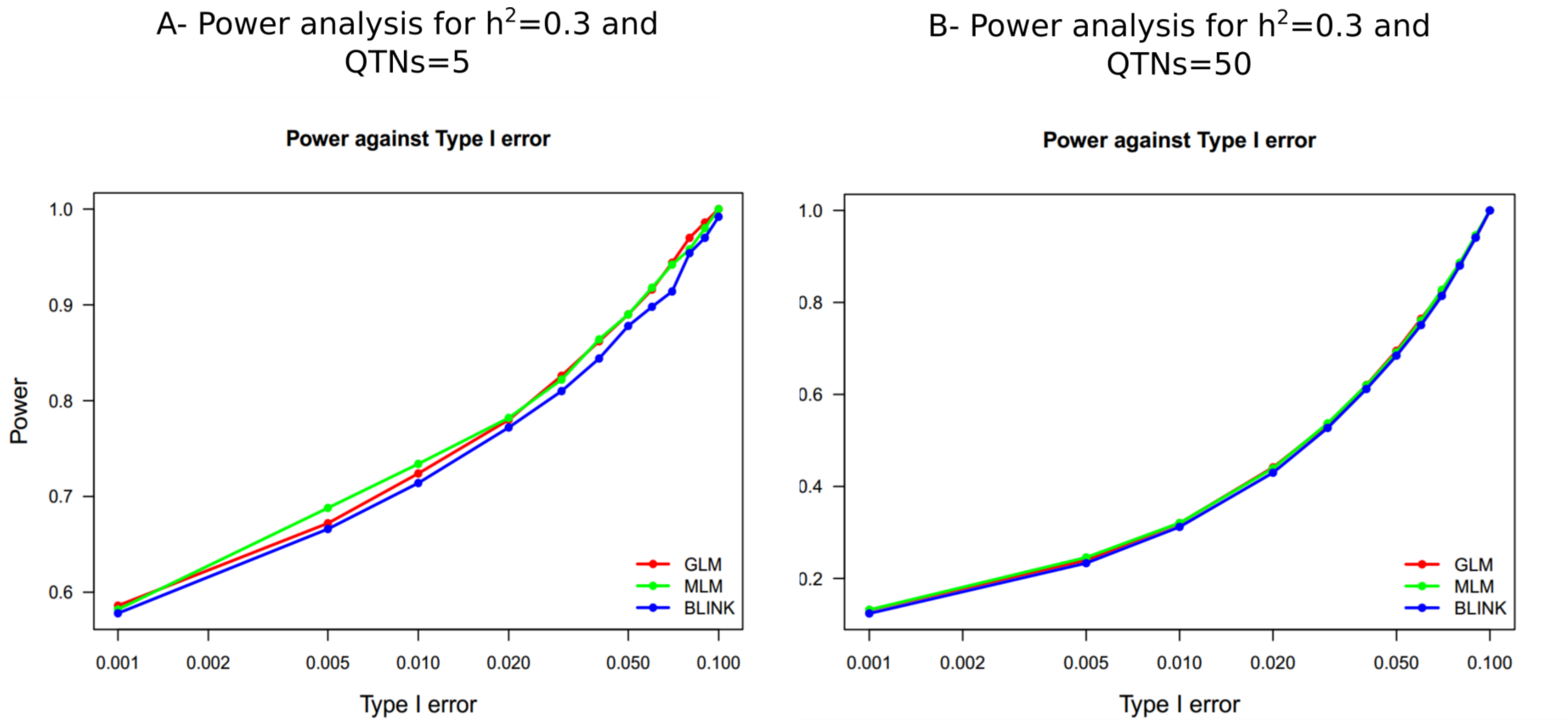

Fig.S6 Comparison between GWAS methods (GLM = red, MLM = green, BLINK multi-locus = blue) on statistical power against Type I error. The comparison was based on a simulated trait with a heritability of 30% using the total great tit dataset containing 342 individuals and 248,325 SNPs under two scenarios: The simulated trait was controlled by A) 5 Quantitative Trait Nucleotides (QTN) and B) 50 Quantitative Trait Nucleotides (QTN). Power was calculated as the proportion of QTN detected. Type I error was calculated as the proportion of tests with false positives. The simulations were replicated 100 times.

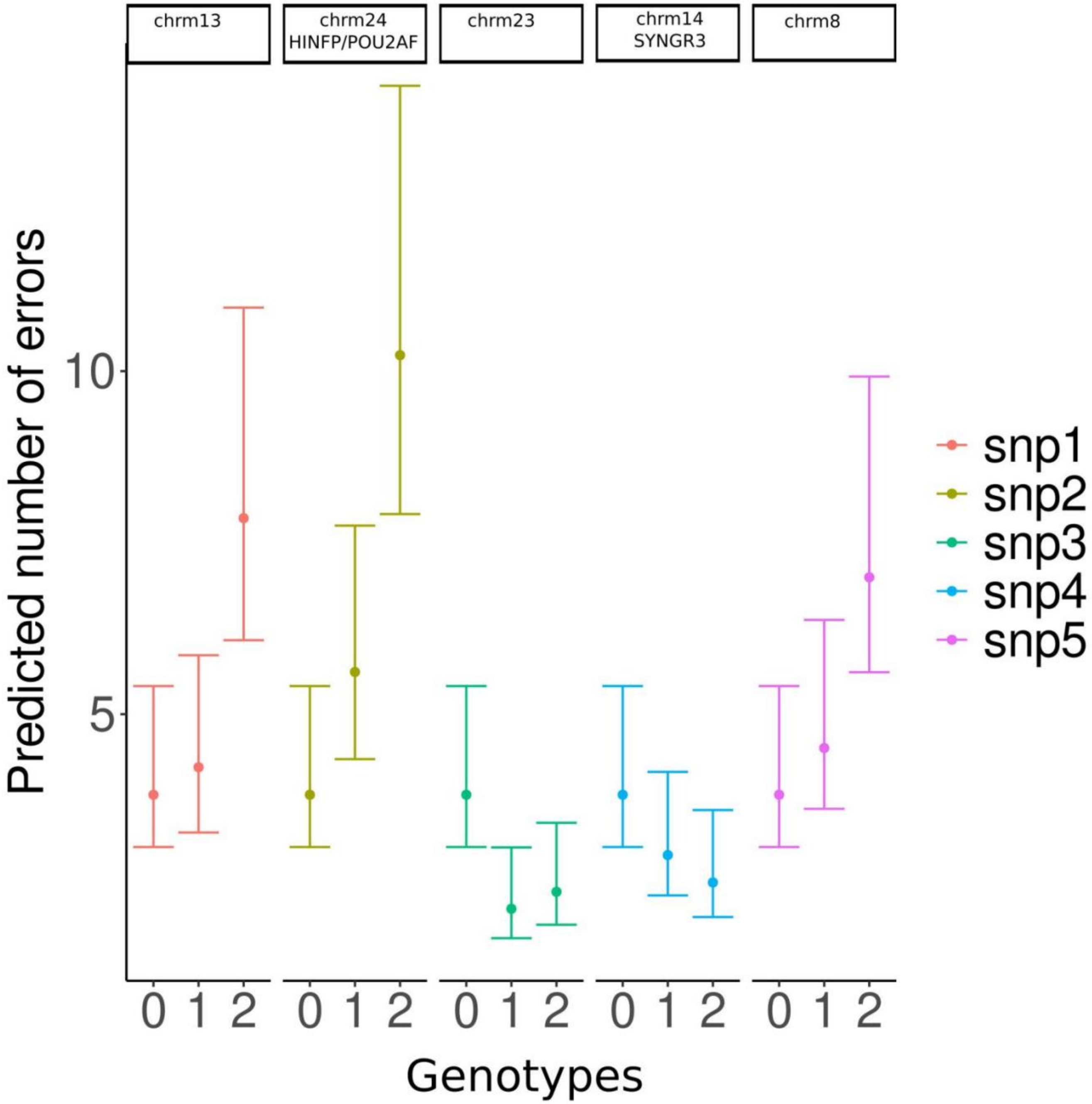

Fig.S7 Relationship and 95% credible interval between genotypes and the predicted number of errors for significant SNPs. We use here model predictions rather than raw values to illustrate the effects of the SNPs. Genotypes are coded as 0, 1, and 2 for homozygous alternative alleles, heterozygotes, and homozygous reference alleles, respectively. Note: There were no significant SNPs associated with latency to escape.

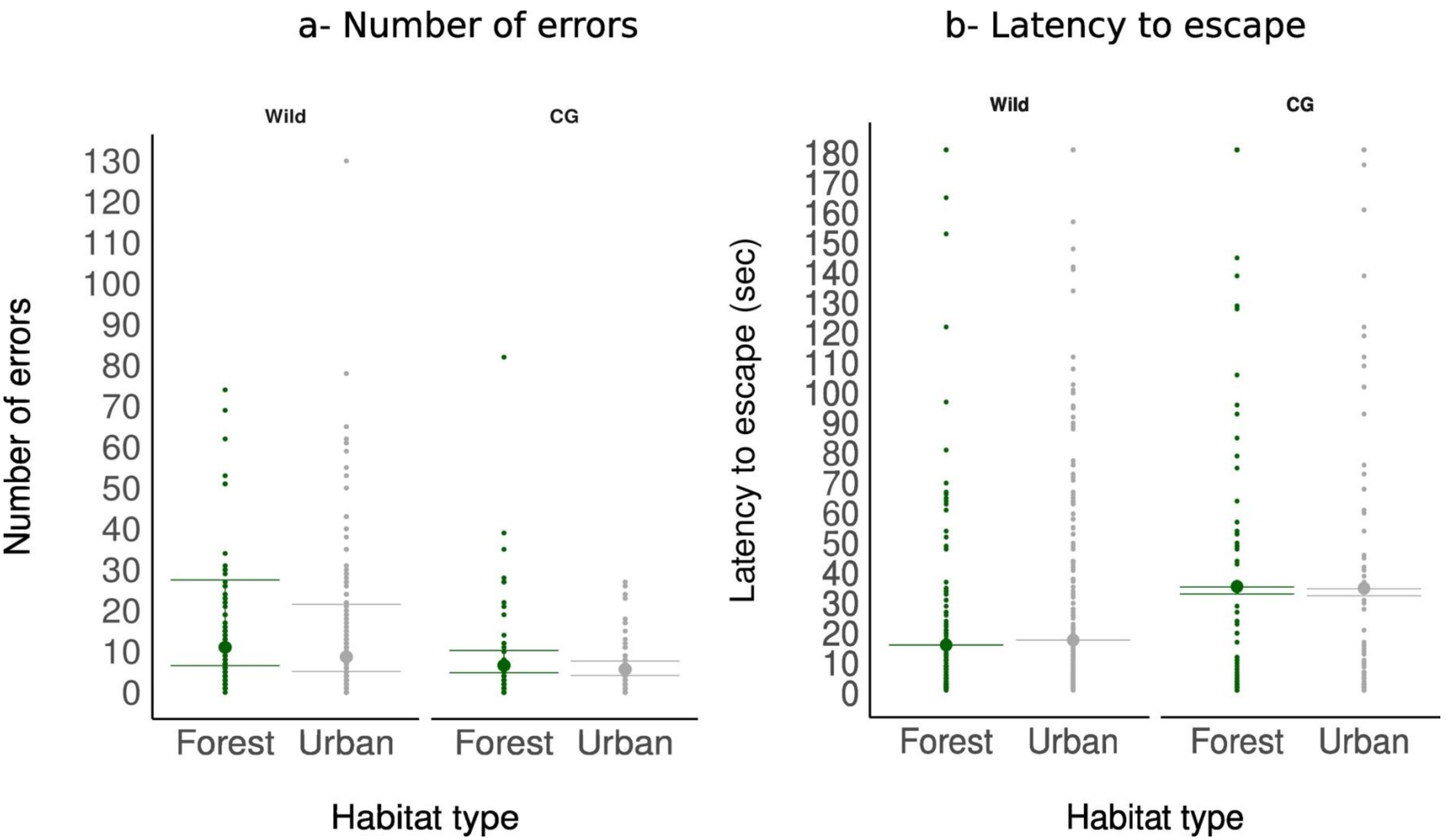

Fig.S8 The effect of habitat type (forest = green vs. urban = grey) and associated 95% credible intervals (horizontal lines) on the a) number of errors and b) latency to escape in wild (left panel within each plot) and common garden (CG; left panel within each plot) contexts. Figure visualizes effects shown in Figure 2 of main text for the full scale of the raw data. Number of errors and latency to escape are expressed on the observed data scale. The large dots correspond to the average predicted effect, while the small dots correspond to the raw data.

Table S1. Leave-one-out cross validation information criterion (LOOIC) and their corresponding standard errors (in square brackets) from models comparing interaction term structures for both the A) number of errors and B) latency to escape in wild and common garden contexts (see main text where each model number is fully described). Instances where ISA* or habitat* are followed by (trial+sex+age) indicates that two-way interactions were fit with ISA or habitat and all these terms.

Model structure:	A) ERRORS	B) LATENCY
Wild	*Model 1 - ISA*	*Model 3 - ISA*
No interactions	2871.21 [50.40]	**3257.94 [62.71]**
ISA*trial + others fixed effects	**2866.39 [50.19]**	3259.05 [62.63]
ISA*(trial+sex+age) + others fixed effects	2867.58 [49.88]	3262.45 [62.41]
Wild	*Model 1 - habitat*	*Model 3 - habitat*
No interactions	**2867.30 [49.97]**	**3257.45 [62.59]**
Habitat*trial+ others fixed effects	2870.49 [50.22]	3261.41 [62.44]
Habitat*(trial+sex+age) + others fixed effects	2874.07 [50.36]	3264.12 [62.34]
Common garden	*Model 2 - ISA*	*Model 4 - ISA*
No interactions	**822.55 [31.22]**	**1269.70 [36.86]**
ISA ISA*trial+ others fixed effects	824.78 [31.56]	1274.65 [36.50]
ISA*(trial+sex+age) + others fixed effects	826.33 [31.39]	1276.14 [36.20]
Common garden	*Model 2 - habitat*	*Model 4 - habitat*
No interactions	**823.70 [31.37]**	**1269.66 [36.94]**
Habitat*trial+ others fixed effects	823.88 [31.43]	1273.33 [36.42]
Habitat*(trial+sex) + others fixed effects	826.42 [31.66]	1274.85 [36.32]

Table S2. Re-analysis of the a) number of errors and b) latency to escape in the common garden context when using a censored regression model that also includes data from individuals that did not escape the task (censored at 180 seconds). Results are qualitatively similar to those presented in Table 1 of the main text where individuals that did not escape were excluded.

**COMMON GARDEN**	**a) ERRORS:** N = 193 obs, 72 ind					**b) LATENCY:** N = 193 obs, 72 ind				
Fixed effects	Median	Mean	*CI*		pdirection	Median	Mean	*CI*		pdirection
Intercept	2.32	2.33	0.23	4.55	0.98	4.74	4.76	1.40	7.88	0.99
ISA	0.22	0.23	-0.23	0.70	0.83	0.07	0.08	-0.93	1.18	0.55
Sex (male)	-0.14	-0.14	-0.97	0.74	0.64	0.31	0.32	-0.32	0.95	0.83
Time of day	-0.03	-0.03	-0.24	0.17	0.62	-0.08	-0.09	-0.32	0.95	0.70
Trial (2)	-0.36	-0.36	-0.85	0.11	0.93	-0.49	-0.49	-1.19	0.23	0.91
Trial (3)	-0.45	-0.45	-0.92	0.02	0.97	**-2.09**	**-2.09**	**-2.82**	**-1.31**	**1**
Random effects										
Individual ID (V_I)	0.22	0.24	<0.001	0.56		0.30	0.38	<0.001	1.02	
Origin nest ID (V_{NO})	0.07	0.12	<0.001	0.39		0.06	0.13	<0.001	0.52	
Foster nest ID (V_{NF})	0.04	0.10	<0.001	0.37		0.04	0.11	<0.001	0.43	
Aviary ID (V_{AV})	0.17	0.26	<0.001	0.78		0.05	0.13	<0.001	0.48	
Residual (V_R)	1.39	1.43	0.85	2.08		3.75	3.77	2.75	4.85	
Repeatability (*R*)	**0.29**	**0.35**	**0.05**	**0.61**		**0.14**	**0.15**	**0.002**	**0.34**	

Table S3. Model comparison to Table 1 in main text when examining the site-level proportion ISA effect (impervious surface area; 100m) instead of the habitat type (forest vs. urban) effect. Posterior median and mean, credible intervals (CI), and pdirection (probability effect is in the same direction as median) for fixed and random effects from separate 1) wild and 2) common garden contexts evaluating the a) number of errors and b) latency to escape in a motor detour task. The number of errors was fit with negative binomial generalized linear mixed-effect models and the latency to escape was fit with a truncated lognormal mixed-effect models. The number of observations (obs), individuals (ind), and repeated individual measures are shown for each context and trait. Estimates whose credible intervals do not cross 0 are bolded.

**1) WILD**	**a) ERRORS:** N = 442 obs, 380 ind (56 ind – 2 trials, 10 ind – 3 trials)					**b) LATENCY:** N = 439 obs, 377 ind (56 ind – 2 trials, 10 ind – 3 trials)				
Fixed effects	Median	Mean	*CI*		pdirection	Median	Mean	*CI*		pdirection
Intercept	**2.28**	**2.28**	**0.48**	**4.11**	**0.99**	**2.50**	**2.49**	**0.39**	**4.64**	**0.99**
ISA	-0.40	-0.40	-1.00	0.26	0.91	0.14	0.14	-0.62	0.88	0.67
Sex (male)	0.09	0.09	-0.14	0.32	0.78	0.09	0.09	-0.16	0.34	0.76
Age (juvenile)	0.04	0.04	-0.21	0.29	0.62	0.08	0.08	-0.20	0.35	0.70
Julian date	<0.001	<0.001	-0.01	0.01	0.63	<0.001	<0.001	-0.01	0.01	0.55
Time of day	-0.03	-0.03	-0.09	0.03	0.82	-0.04	-0.04	-0.11	0.04	0.83
Year (2022)	-0.07	-0.07	-0.32	0.18	0.70	-0.09	-0.09	-0.37	0.19	0.73
Year (2023)	-0.07	-0.07	-0.38	0.23	0.69	-0.01	-0.01	-0.35	0.32	0.52
Blood (yes)	-0.20	-0.20	-0.55	0.14	0.87	-0.23	-0.23	-0.63	0.15	0.88
Trial (2)	-0.16	-0.16	-0.59	0.28	0.76	0.10	0.10	-0.29	0.50	0.69
Trial (3)	-0.29	-0.29	-1.24	0.69	0.72	-0.57	-0.57	-1.39	0.27	0.91
ISA*Trial (2)	**1.24**	**1.24**	**0.34**	**2.14**	**1.00**					
ISA*Trial (3)	-0.14	-0.12	-2.18	1.94	0.55					
Random effects										
Individual ID (V_I)	**0.49**	**0.49**	**0.31**	**0.69**		0.28	0.29	<0.001	0.61	
Site ID (V_{SITE})	0.02	0.05	<0.001	0.18		0.04	0.07	<0.001	0.26	
Residual (V_R)	**0.67**	**0.70**	**0.46**	**0.95**		**1.21**	**1.22**	**0.85**	**1.60**	
Repeatability (*R*)	**0.4**	**0.4**	**0.25**	**0.55**		0.18	0.19	<0.001	0.38	
**2) COMMON GARDEN**	**a) ERRORS:** N = 153 obs, 72 ind (54 ind – 2 trials, 32 ind – 3 trials)					**b) LATENCY:** N = 153 obs, 72 ind (54 ind – 2 trials, 32 ind – 3 trials)				
Fixed effects	Median	Mean	*CI*		pdirection	Median	Mean	*CI*		pdirection
Intercept	1.05	1.05	-0.78	2.96	0.86	3.04	3.01	-0.46	6.73	0.95
ISA	-0.21	-0.22	-0.99	0.57	0.71	-0.02	-0.02	-1.14	1.14	0.52
Sex (male)	0.20	0.20	-0.22	0.64	0.82	0.30	0.31	-0.41	1.00	0.81
Time of day	0.06	0.06	-0.12	0.24	0.74	<0.001	<0.001	-0.35	0.35	0.50
Trial (2)	-0.28	-0.28	-0.70	0.17	0.90	0.13	0.13	-0.73	1.05	0.63
Trial (3)	-0.43	-0.43	-0.87	0.02	0.97	**-1.41**	**-1.43**	**-2.28**	**-0.54**	**1.00**
Random effects										
Individual ID (V_I)	0.21	0.23	<0.001	0.51		0.13	0.21	<0.001	0.69	
Origin nest ID (V_{NO})	0.04	0.08	<0.001	0.28		0.04	0.09	<0.001	0.55	
Foster nest ID (V_{NF})	0.03	0.08	<0.001	0.33		0.05	0.12	<0.001	0.50	
Aviary ID (V_{AV})	0.11	0.18	<0.001	0.57		0.31	0.50	<0.001	1.58	
Residual (V_R)	**0.63**	**0.63**	**0.36**	**0.87**		**2.66**	**2.73**	**1.80**	**3.80**	
Repeatability (*R*)	**0.35**	**0.37**	**0.06**	**0.71**		**0.22**	**0.24**	**0.02**	**0.51**	

Table S4. Model comparison to Table 1 and Table S3 when only including the first trial for wild birds. Posterior median and mean, credible intervals (CI), and pdirection (probability effect is in the same direction as median) for fixed and random effects in models using the 1) habitat effect and 2) ISA effect while evaluating the a) number of errors and b) latency to escape in a motor detour task. The number of errors was fit with negative binomial generalized linear mixed-effect models and the latency to escape was fit with a truncated lognormal mixed-effect models. The number of observations (obs), individuals (ind) are shown for each context and trait. Estimates whose credible intervals do not cross 0 are bolded.

**1) Habitat**	**a) ERRORS:** N = 442 obs, 380 ind (60 ind – 2 trials, 10 ind – 3 trials)					**b) LATENCY:** N = 439 obs, 377 ind (56 ind – 2 trials, 10 ind – 3 trials)				
Fixed effects	Median	Mean	*CI*		pdirection	Median	Mean	*CI*		pdirection
Intercept	1.82	1.79	-0.41	3.91	0.94	1.89	1.90	-0.38	4.39	0.94
Habitat	-0.25	-0.24	-0.91	0.45	0.82	0.09	0.10	-0.76	0.90	0.61
Sex (male)	0.07	0.07	-0.16	0.30	0.72	0.11	0.11	-0.15	0.39	0.80
Age (juvenile)	0.01	0.01	-0.24	0.26	0.54	0.06	0.06	-0.21	0.37	0.66
Julian date	0.01	0.01	<0.001	0.02	0.89	<0.001	<0.001	-0.01	0.02	0.69
Time of day	-0.02	-0.02	-0.08	0.05	0.71	-0.02	-0.02	-0.10	0.06	0.68
Year (2022)	-0.03	-0.03	-0.28	0.23	0.58	-0.04	-0.04	-0.31	0.28	0.61
Year (2023)	-0.16	-0.16	-0.46	0.14	0.86	-0.01	-0.01	-0.37	0.36	0.53
Blood (yes)	-0.38	-0.38	-0.79	0.01	0.97	-0.24	-0.24	-0.70	0.24	0.83
Random effects										
Site ID (V_{SITE})	0.05	0.09	<0.001	0.28		0.05	0.08	<0.001	0.29	
Residual (V_R)	**1.59**	**1.60**	**1.26**	**1.96**		**1.5**	**1.5**	**1.27**	**1.75**	
**2) ISA**										
Fixed effects	Median	Mean	*CI*		pdirection	Median	Mean	*CI*		pdirection
Intercept	1.92	1.92	-0.15	3.94	0.97	1.72	1.73	-0.75	4.07	0.92
ISA	-0.43	-0.42	-1.02	0.24	0.92	0.14	0.14	-0.53	0.86	0.70
Sex (male)	0.06	0.07	-0.16	0.29	0.71	0.11	0.11	-0.13	0.38	0.81
Age (juvenile)	0.02	0.02	-0.22	0.25	0.56	0.07	0.07	-0.22	0.35	0.68
Julian date	0.01	0.01	-0.01	0.02	0.85	<0.001	<0.001	-0.01	0.02	0.73
Time of day	-0.02	-0.02	-0.08	0.05	0.69	-0.02	-0.02	-0.10	0.06	0.66
Year (2022)	-0.02	-0.02	-0.27	0.24	0.55	-0.04	-0.04	-0.32	0.28	0.60
Year (2023)	-0.15	-0.15	-0.44	0.15	0.84	-0.01	-0.01	-0.35	0.36	0.53
Blood (yes)	-0.37	-0.38	-0.79	0.01	0.97	-0.23	-0.23	-0.69	0.25	0.83
Random effects										
Site ID (V_{SITE})	0.02	0.05	<0.001	0.19		0.04	0.08	<0.001	0.29	
Residual (V_R)	**1.58**	**1.59**	**1.26**	**1.94**		**1.50**	**1.50**	**1.26**	**1.75**	

Table S5. Posterior median and mean, credible intervals (CI) for fixed and random effects evaluating the association between the five significant SNPs and number of errors in a motor detour task for wild birds. The number of errors was fit with negative binomial generalized linear mixed-effect models. Fixed- and random-effect estimates whose credible intervals do not cross 0 or are greater than 0.001, respectively, are bolded. The five SNPs fitted as fixed effects were the ones that were significant in the GWAS analysis. Note that the sum of the individual SNP effects does not equal the total SNP effect because the sum of the medians differs from the median of the sums.

Fixed effects	Median	Mean	CI	
Intercept	**0.97**	**0.97**	**0.44**	**1.48**
SNP1 (Chrom 8: gene unknown)	**0.26**	**0.26**	**0.07**	**0.45**
SNP2 (Chrom 13 gene: LOC107210618/ LOC117245099)	**0.55**	**0.55**	**0.32**	**0.77**
SNP3 (Chrom 14 gene: SYNGR3)	**-0.53**	**-0.53**	**-0.82**	**-0.24**
SNP4 (Chrom 23: gene unknown)	**-0.22**	**-0.22**	**-0.40**	**-0.05**
SNP5 (Chrom 24 gene: POU2AF2/ HINFP)	**0.31**	**0.31**	**0.14**	**0.48**
Random effects				
SNP1	0.03	0.03	<0.001	0.08
SNP2	**0.10**	**0.10**	**0.03**	**0.19**
SNP3	**0.06**	**0.06**	**0.01**	**0.13**
SNP4	0.03	0.03	<0.001	0.07
SNP5	0.06	0.06	<0.001	0.12
Hmatrix (V_A_nonSNP)	0.10	0.13	<0.001	0.34
Individual ID (V_{PE})	0.18	0.18	<0.001	0.39
Site ID (V_{SITE})	0.07	0.11	<0.001	0.36
Residual (V_R)	**0.70**	**0.71**	**0.48**	**0.97**
Proportion of variance explained				
Total SNPs	**0.21**	**0.21**	**0.12**	**0.30**
SNP1	0.02	0.02	0.00	0.05
SNP2	**0.07**	**0.07**	**0.02**	**0.13**
SNP3	0.04	0.05	<0.001	0.09
SNP4	0.02	0.02	<0.001	0.05
SNP5	0.04	0.04	<0.001	0.09
Individual ID (V_{PE})	0.13	0.13	<0.001	0.28
Site ID (V_{SITE})	0.05	0.07	<0.001	0.22
Residual (V_R)	**0.50**	**0.50**	**0.35**	**0.65**
Heritability (h^2_nonSNP)	0.07	0.09	<0.001	0.24
